# Supplementary material for: The impact of vitamin D deficiency on some biochemical parameters and clinical outcome in Palestinian pregnant women during the first trimester
Source: PLoS One. 2023 Mar 30;18(3):e0283392. doi: 10.1371/journal.pone.0283392 (PMC10062660; doi:10.1371/journal.pone.0283392)
Supplement: Attachment — Submitted filename: Response to Reviewers.docx [file pone.0283392.s001.docx]

**Journal Requirements and reviewer comments:**

| **Suggestions** | 1. Please ensure that your manuscript meets PLOS ONE's style requirements, including those for file naming. The PLOS ONE style templates can be found at  https://journals.plos.org/plosone/s/file?id=wjVg/PLOSOne_formatting_sample_main_body.pdf and  https://journals.plos.org/plosone/s/file?id=ba62/PLOSOne_formatting_sample_title_authors_affiliations.pdf |
| --- | --- |
| **Corrections** | We would like to thanks supporting us with valuable comments, we address these additional requirements according to the PLOS ONE style templates in the provided links and highlighting in red color. |
| **Suggestions** | 2. Please provide additional details regarding participant consent. In the ethics statement in the Methods and online submission information, please ensure that you have specified what type you obtained (for instance, written or verbal, and if verbal, how it was documented and witnessed). If your study included minors, state whether you obtained consent from parents or guardians. If the need for consent was waived by the ethics committee, please include this information. |
| **Corrections** | We respect your comments and specified in the ethics statement in the Methods that we obtained written informed consent form.  For ethical consideration, the necessary approval to conduct this study was obtained from Helsinki committee with ethical approval number PHRC/HC/559/19 in the Gaza Strip and approval letter signed from the Palestinian Ministry of Health (MOH). All participants freely signed the written informed consent form of the study and freely participated in the study. |
| **Suggestions** | 3. We noticed you have some minor occurrence of overlapping text with the following previous publication(s), which needs to be addressed: - 10.4103/0976-9668.160021  - https://doi.org/10.1089/thy.2011.0087 - https://doi.org/10.1007/s00404-018-5018-8 In your revision ensure you cite all your sources (including your own works), and quote or rephrase any duplicated text outside the methods section. Further consideration is dependent on these concerns being addressed. |
| **Corrections** | We cite all of the sources including our works, and rephrase any duplicated text.  - HCG has a stimulatory impact on thyroid hormone synthesis and release by binding to the TSH receptor on thyrocytes.  - Greater fT4 values are often associated with higher TBG concentrations in blood samples, whereas lower fT4 values are likely to be associated with lower albumin concentrations in serum.  - Anti-TPO is associated with a higher risk of pregnancy complications such as placental abruption, miscarriage preterm delivery and pregnancy-induced hypertension |
| **Suggestions** | 4. In your Data Availability statement, you have not specified where the minimal data set underlying the results described in your manuscript can be found. PLOS defines a study's minimal data set as the underlying data used to reach the conclusions drawn in the manuscript and any additional data required to replicate the reported study findings in their entirety. All PLOS journals require that the minimal data set be made fully available. For more information about our data policy, please see http://journals.plos.org/plosone/s/data-availability.  "Upon re-submitting your revised manuscript, please upload your study’s minimal underlying data set as either Supporting Information files or to a stable, public repository and include the relevant URLs, DOIs, or accession numbers within your revised cover letter. For a list of acceptable repositories, please see http://journals.plos.org/plosone/s/data-availability#loc-recommended-repositories. Any potentially identifying patient information must be fully anonymized.  Important: If there are ethical or legal restrictions to sharing your data publicly, please explain these restrictions in detail. Please see our guidelines for more information on what we consider unacceptable restrictions to publicly sharing data: http://journals.plos.org/plosone/s/data-availability#loc-unacceptable-data-access-restrictions. Note that it is not acceptable for the authors to be the sole named individuals responsible for ensuring data access.  We will update your Data Availability statement to reflect the information you provide in your cover letter. |
| **Corrections** | We add in the title page the following data availability statement: All relevant data are within the manuscript and fully available without restriction. |
| **Reviewer Suggestions** | 1. Is the manuscript technically sound, and do the data support the conclusions?  The manuscript must describe a technically sound piece of scientific research with data that supports the conclusions. Experiments must have been conducted rigorously, with appropriate controls, replication, and sample sizes. The conclusions must be drawn appropriately based on the data presented.  Reviewer #1: Yes  Reviewer #2: Yes |
| **Corrections** | We thank both of the reviewers for their kind comment and we critically read and revised our manuscript and highlighting the changes in red color. |
| **Reviewer Suggestions** | 2. Has the statistical analysis been performed appropriately and rigorously?  Reviewer #1: Yes  Reviewer #2: Yes |
| **Corrections** | We made the following tests were applied; ANOVA test, the significance of difference was checked by one-way ANOVA test. Chi-square test was used to determine how the proportions differed. Spearman correlation coefficient and independent-samples t-test were also used to calculate the variance in means across groups. |
| **Reviewer Suggestions** | 3. Have the authors made all data underlying the findings in their manuscript fully available?  The [PLOS Data policy](http://www.plosone.org/static/policies.action#sharing) requires authors to make all data underlying the findings described in their manuscript fully available without restriction, with rare exception (please refer to the Data Availability Statement in the manuscript PDF file). The data should be provided as part of the manuscript or its supporting information, or deposited to a public repository. For example, in addition to summary statistics, the data points behind means, medians and variance measures should be available. If there are restrictions on publicly sharing data—e.g. participant privacy or use of data from a third party—those must be specified.  Reviewer #1: Yes  Reviewer #2: Yes |
| **Corrections** | We add in the title page the following data availability statement: All relevant data are within the manuscript and fully available without restriction. |
| **Reviewer Suggestions** | 4. Is the manuscript presented in an intelligible fashion and written in standard English?  PLOS ONE does not copyedit accepted manuscripts, so the language in submitted articles must be clear, correct, and unambiguous. Any typographical or grammatical errors should be corrected at revision, so please note any specific errors here.  Reviewer #1: Yes  Reviewer #2: Yes |
| **Corrections** | Our collogue critically read our manuscript and revised the English and typo as required. |
| **Reviewer Suggestions** | 5. Review Comments to the Author  Please use the space provided to explain your answers to the questions above. You may also include additional comments for the author, including concerns about dual publication, research ethics, or publication ethics. (Please upload your review as an attachment if it exceeds 20,000 characters)  Reviewer #1: Thyroid health during pregnancy is very important both mother and fetus. Although n number is low it is found relationship between Vitamine D levels and TSH and FT4. This study has relevant results for clinical purposes.  Reviewer #2: The subject is relevant and deserves to be encouraged. The point is the form of your tables. The tables should be scientific. Did you follow ethical procedures? Thank you for trusting us to review the articles |
| **Corrections** | We would like to thanks Reviewer #1 for spending their valuable time in revision our manuscript and supporting us with valuable comments. Thank for your kind word that describing the important and clinical utility of vitamin D deficiency on some biochemical parameters and clinical outcome in Palestinian pregnant women during the first trimester and the important of thyroid for mother and child.  We respect your decision concerning our manuscript. However, we critically re-read the manuscript and re-revised the English and typo and we re-standardized and re-format all tables in our manuscript |
|  | We would like to thanks Reviewer #2 for spending their valuable time in revision our manuscript and supporting us with valuable comments. We respect your decision concerning our manuscript and followed your advice and we critically re-read the manuscript and re-revised the English and typo and re-standardized and re-format all tables in our manuscript.  We respect your comments and specified in the ethics statement in the Methods that we obtained written informed consent form.  For ethical consideration, the necessary approval to conduct this study was obtained from Helsinki committee with ethical approval number PHRC/HC/559/19 in the Gaza Strip and approval letter signed from the Palestinian Ministry of Health (MOH). All participants freely signed the written informed consent form of the study and freely participated in the study. |
